# Supplementary material for: A UMLS-based spell checker for natural language processing in vaccine safety
Source: BMC Med Inform Decis Mak. 2007 Feb 12;7:3. doi: 10.1186/1472-6947-7-3 (PMC1805499; doi:10.1186/1472-6947-7-3)
Supplement: Additional file 13 — RAP application directory. Sets up directory for RAP (RDF application for PHP) source code [file 1472-6947-7-3-S13.gz › rap/doc/phpdoc/syntax/NTripleSerializer.html]

xml version="1.0" encoding="iso-8859-1"?


Docs For Class NTripleSerializer


## Class NTripleSerializer

Description

Description |
Vars (details)
| Methods (details)

PHP N-Triple Serializer

This class serialises models to N-Triple Syntax.

**History:**

- 11-15-2003 Initial version

- access: public
- version: V0.9.1
- author: Daniel Westphal <mail@d-westphal.de>

Located in /syntax/NTripleSerializer.php (line 28)

```
Object
   |
   --NTripleSerializer
```

Variable Summary

Description |
Vars (details)
|
Methods (details)

mixed
$debug

mixed
$model

mixed
$res

Method Summary

Description |
Vars (details)
|
Methods (details)

NTripleSerializer
NTripleSerializer
()

boolean
saveAs
(object MemModel &$model, string $filename)

string
&serialize
(mixed &$m, object Model $model)

Variables

Description |
Vars (details)
|
Methods (details)

mixed
$debug
(line 30)

mixed
$model
(line 31)

mixed
$res
(line 32)

Methods

Description |
Vars (details)
Methods (details)

Constructor NTripleSerializer (line 43)

Constructor

- access: public

NTripleSerializer

NTripleSerializer
()

saveAs (line 104)

Serializes a model and saves it into a file.

Returns FALSE if the model couldn't be saved to the file.

- access: public

boolean

saveAs
(object MemModel &$model, string $filename)

- object MemModel
  $model
- string
  $filename

serialize (line 54)

Serializes a model to N Triple syntax.

- access: public

string

&serialize
(mixed &$m, object Model $model)

- object Model
  $model

#### Inherited Methods


Inherited From Object

> Object::toString()

Documentation generated on Thu, 7 Jul 2005 13:42:18 +0200 by phpDocumentor 1.3.0RC3
